# Autoimmune response to PARP and BRCA1/BRCA2 in cancer

**DOI:** 10.18632/oncotarget.3428

**Published:** 2015-03-24

**Authors:** Qing Zhu, Su-Xia Han, Cong-Ya Zhou, Meng-Jiao Cai, Li-Ping Dai, Jian-Ying Zhang

**Affiliations:** ^1^ Department of Oncology, The First Affiliated Hospital of Xi'an Jiaotong University Medical Center, Xi'an, Shaanxi, P.R. China; ^2^ Department of Biological Sciences, The University of Texas at El Paso, El Paso, Texas, USA

**Keywords:** autoantibody, DNA repair enzyme poly (ADP-Ribose) polymerase 1 (PARP1)/BRCA1/BRCA2, synthetic lethality, tumor-associated antigen (TAA), autoimmune

## Abstract

**Purpose:**

To determine the role of autoantibodies to PARP1 and BRCA1/BRCA2 which were involved in the synthetic lethal interaction in cancer.

**Methods:**

Enzyme-Linked Immunosorbent Assay (ELISA) was used to detect autoantibodies to PARP1 and BRCA1/BRCA2 in 618 serum samples including 131 from breast cancer, 94 from lung cancer, 34 from ovarian cancer, 107 from prostate cancer, 76 from liver cancer, 41 from pancreatic cancer and 135 from normal individuals. The positive sera with ELISA were confirmed by Western blot. Immunohistochemistry was used to examine the expression of PARP1 and BRCA1/BRCA2 in breast cancer.

**Results:**

Autoantibody frequency to PARP1, BRCA1, and BRCA2 in cancer varied from 0% to 50%. When the sera from cancer patients were tested for the presence of autoantibodies to PARP1 and BRCA1/BRCA2, the autoantibody responses slightly decreased and the positive autoantibody reactions varied from 0% to 50.0%. This was significantly higher autoantibody responses to PARP1 and BRCA1/BRCA2 (especially to PARP1 and BRCA1) in ovarian cancer and breast cancer compared to normal control sera (*P* < 0.001 and *P* < 0.01). Immunohistochemistry indicated that Pathology Grade at diagnosis to PARP1 expression in breast cancer was different (*P* < 0.05).

**Conclusions:**

Different cancers have different profiles of autoantibodies. The autoantibodies to proteins involving the synthetic lethal interactions would be novel serological biomarker in some selective cancers.

## INTRODUCTION

Many studies have demonstrated that dysfunction of the tumor suppressor genes, either BRCA1 or BRCA2, is synthetically lethal with inhibition of the DNA repair enzyme poly (ADP-Ribose) Polymerase 1 (PARP1) [1–3]. BRCA1 or BRCA2 deficient cells are not only inhibited by gene silencing of PARP1 but are also profoundly sensitive to potent small molecular PARP inhibitors [4]. Synthetic lethality therefore provides a conceptual framework for the development of cancer-specific cytotoxic agents. But it has been over 15 years since that frame work was proposed and synthetic lethal therapies have largely failed to deliver [5]. Gene-gene interaction, including synthetic lethal interactions that are discovered *in vitro* cell-culture experiments, will ultimately need to be validated *in vivo*. It seems likely that some gene-gene interaction will be highly robust, whereas others might be valid only in specific cells or under specific experimental therapy conditions. It has been reported that a potent PARP inhibitor was not only administered safely but also elicited significant responses in BRCA mutation carriers with breast, ovarian or prostate cancers. In addition, elevated PARP1 expression in lung cancer has also been proposed as a predictive biomarker of PARP inhibitor response [6].

Intracellular proteins involved in carcinogenesis have been shown to provoke autoantibody responses, therefore autoantibodies can be used as probes in immunoproteomics to isolate, identify, and characterize potential tumor-associated antigens (TAAs) [7]. Although the mechanisms underlying the production of autoantibody in cancer patients are not completely understood, emerging evidence indicates that most TAAs are cellular proteins whose aberrant regulation of function could be linked to malignancy. In cancer immunodiagnosis, the major task ahead is the continuing identification of TAAs, and the challenging problem is the separation of tumor-associated from non-tumor-associated antigens, because autoantibodies to other cellular antigens can be present before appearance of new antibodies occurring with malignancy [8]. So it is important to validate a candidate TAA by testing not only with cancer sera but also with sera from patients with pre-cancer conditions and with sera from other autoimmune disorders. Tan and his colleagues, for the first time, reported the immunogenic and autoantibodies to PARP1 in human sera and the presence of autoantibodies to PARP1were in the sera from cancer patients with Paraneoplastic Neurologic Syndrome [9]. In addition, our previous studies have also demonstrated that using a panel of recombinant TAAs could enhance the sensitivity and specificity of autoantibody detection in cancer [10–13]. Therefore, the molecular identification and characterization of synthetic lethality in cancer will also contribute to our understanding the role of autoantibodies in malignant transformation, thereby providing attractive candidates for both early diagnosis and targeted therapies. This study tested and validated the usefulness of the concept of synthetic lethality to further understand the functions of some oncogenes and the mechanism of autoantibody production in cancer, which might have potential application in cancer immunodiagnosis and cancer treatment.

## RESULTS

### Frequency and titers of autoantibodies to three TAAs (PARP1, BRCA1 and BRCA2) in cancer

**Figure 1:**
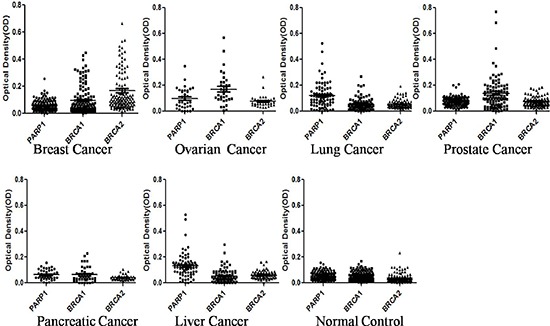
Titer of autoantibodies to PARP1, BRCA1 and BRCA2 in sera from patients with breast, lung, ovarian, prostate, liver and pancreatic cancers, as well as sera from normal controls The range of autoantibody titers to each of three partners is expressed as optical density (OD) obtained from ELISA. The high titer of reactivity in cancer sera and the distinct difference between cancer and normal controls are demonstrated in this figure. The Y-axis represents the OD values. The X-axis represents the synthetic lethality partner of three TAAs including PARP1, BRCA1 and BRCA2.

In this study, purified recombinant proteins (PARP1, BRCA1 and BRCA2) were commercially purchased, and used as coating antigens. Sera from patients with cancer and normal controls were examined for the presence of autoantibodies to the individual TAA. Table 1 shows the frequency of autoantibodies to three TAAs (PARP1, BRCA1 and BRCA2) in sera from patients with different conditions using ELISA. The sera that were tested included the 131 sera from patients with breast cancer, 94 with lung cancer, 34 with ovarian cancer, 107 with prostate cancer, 76 with liver cancer, and 41 with pancreatic cancer as well as 135 normal controls from healthy individuals. A positive test for antibodies was taken as an absorbance reading above the mean+3SD of the 135 normal control sera. Of a total 131 breast cancer sera analyzed, 15.3% (20/131) was shown to have autoantibody to PARP1, 19.1% (25/131) was shown to have autoantibody to BRCA1, 36.6% (48/131) was shown to BRCA2. In the further analysis, we found that 5.3% (7/131) sera contained autoantibodies to both PARP1 and BRCA1, 7.6% (10/131) to both PARP1 and BRCA2, 4.6% (6/131) to both BRCA1 and BRCA2, and 4.6% (6/131) sera simultaneously to PARP1, BRCA1 and BRCA2 (Table 1). In ovarian cancer sera, 29.4% (10/34) was shown to have autoantibody to PARP1, 50.0% (17/34) was shown to have autoantibody to BRCA1, 5.9% (2/34) was shown to BRCA2, 29.4%(10/34) was shown to PARP1 and BRCA1, 0% (0/34) was shown to PARP1 and BRCA2, 2.9% (1/34) was shown to have autoantibody to BRCA1 and BRCA2, 0% (0/34) was shown to PARP1, BRCA1 and BRCA2. In lung cancer sera, 22.4% (21/94) was shown to have autoantibody to PARP1, 4.3% (4/94) was shown to BRCA1, 1.1% (1/94) was shown to BRCA2, 4.3% (4/94) was shown to PARP1 and BRCA1, and 0% (0/94) was shown to PARP1 and BRCA2. In prostate cancer, autoantibody frequency to PARP1/BRCA1/BRCA2 were 2.8% (3/107), 28.0% (30/107) and 4.7% (5/107), 1.9% (2/107), 0.9% (1/107) and 1.9% (2/107) were shown to have autoantibody to PARP1 and BRCA1, PARP1 and BRCA2, or BRCA1 and BRCA2. In pancreatic cancer, autoantibody frequency to three TAAs (PARP1, BRCA1 and BRCA2) were 0% (0/41), 7.3% (3/41) and 0% (0/41). In liver cancer, autoantibodies frequency to three TAAs were 25.0% (19/76), 5.3% (4/76) and 0% (0/76), 5.3% (4/76), 0% (0/76) and 0% (0/76) were shown to have autoantibody to PARP1 and BRCA1, PARP1 and BRCA2, or BRCA1 and BRCA2, respectively. Higher frequency (*P* < 0.001) of autoantibodies against PARP1 was found in breast, lung, ovarian, and liver cancers. Higher frequency (*P* < 0.001) of autoantibodies to BRCA1 was found in breast cancer, ovarian cancer, and prostate cancer. Higher frequency (*P* < 0.001) of autoantibodies to BRCA2 was found in breast cancer sera. When the cancer sera were tested against a combination of two antigens, higher frequency (*P* < 0.01) of autoantibodies against PARP1 and BRCA1 was found in breast cancer and ovarian cancer. In addition, higher frequency (*P* < 0.01) of autoantibodies to PARP1 and BRCA2 was found only in breast cancer sera. The ranges of antibody titers to these TAAs in different conditions are shown in Figure 1. The high titer reactivity of cancer sera and the distinct difference between cancer and normal controls were also demonstrated. Many cancer sera showed OD values several fold above the cutoff, indicating that autoantibodies response to three TAAs (PARP, BRCA1 and BRCA2) in some cancer patients were quite robust and not just mildly elevated. Positive results were also confirmed by Western blotting assay.

**Table 1:** The same individual serum simultaneously contain autoantibodies to tumor-associated antigens PARP1, BRCA1 and BRCA2 in 618 participants

Sera	NO. Tested	No. and percentage of autoantibodies of the same individual to						
		PARP1	BRCA1	BRCA2	PARP1+BRCA1 PARP1+BRCA2		BRCA1+BRCA2 Three TAAs	
Breast cancer	131	20 (15.3%)***	25 (19.1%)***	48 (36.6%)***	7 (5.3%)**	10 (7.6%)**	6 (4.6%)*	6 (4.6%)*
Lung cancer	94	21 (22.4%)***	4 (4.3%)*	1 (1.1%)	4 (4.3%)*	0 (0%)	0 (0%)	0 (0%)
Ovarian cancer	34	10 (29.4%)***	17 (50.0%)***	2 (5.9%)*	10 (29.4%)***	0 (%)	1 (2.9%)	0 (0%)
Prostate cancer	107	3 (2.8%)	30 (28.0%)***	5 (4.7%)*	2 (1.9%)	1 (0.9%)	2 (1.9%)	0 (0%)
Liver cancer	76	19 (25.0%)***	4 (5.3%)*	0 (0%)	4 (5.3%)*	0 (0%)	0 (0%)	0 (0%)
Pancreatic cancer	41	0 (0%)	3 (7.3%)*	0 (0%)	0 (0%)	0 (0%)	0 (0%)	0 (0%)
Normal control	135	1 (0.7%)	1 (0.7%)	1 (0.7%)	0 (0%)	0 (0%)	0 (0%)	0 (0%)

Three TAAs: PARP1+BRCA1+BRCA2;

*P* Value relative to Nomal controls

* *P* < 0.05

** *P* < 0.01

*** *P* < 0.001.

### Elevated expression of three TAAs PARP1, BRCA1 and BRCA2 in cancer

**Figure 2:**
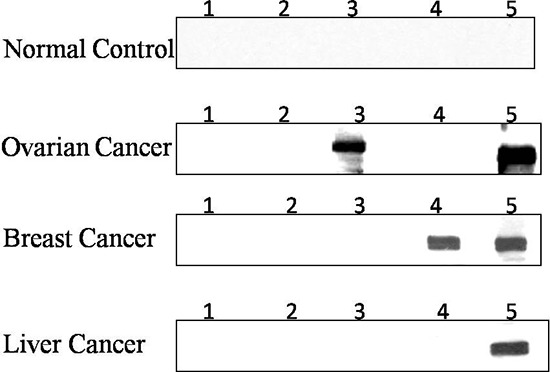
Western blot analysis of three representative cancer sera Each blot represents a duplicate test for antoantibodies against the synthetic lethality partner of either PARP1 and BRCA1 or PARP1 and BRCA2,. Lane 1 and 2, PBS as negative controls; Lane 3, antoantibody against BRCA1; Lane 4, antoantibody against BRCA2; Lane 5, antoantibody against PARP1. Human normal control serum show no reactivity for antoantibodies to any of three synthetic lethality partners; Ovarian cancer serum show a strong reactivity for antoantibodies to BRCA1 and PARP1; Breast cancer serum show reactivity for antoantibodies against BRCA1 and PARP1; Liver cancer serum show a strong reactivity for antoantibody to PARP1.

To confirm the difference of expression of three TAAs including PARP1, BRCA1 and BRCA2 in cancer, ELISA positive cancer sera were also analyzed by Western blotting analysis. As shown in Figure 2, the antibody responses to PARP1, BRCA1, and BRCA2 had strong reactivity in representative cancer sera compared to normal controls. Normal control sera shows no reactivity to these three TAAs.

### Expression of PARP1, BRCA1 and BRCA2 in breast cancer tissues

**Figure 3:**
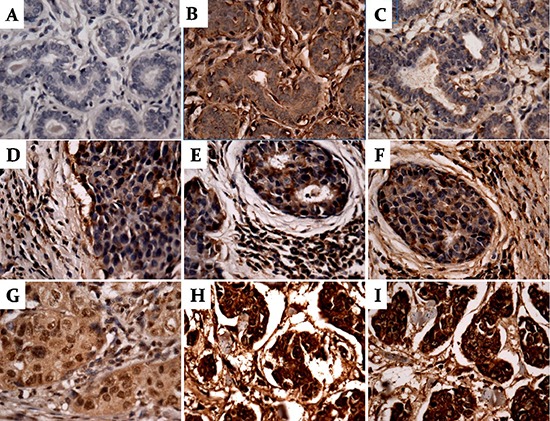
Expression of PARA1, BRCA1 and BRAC2 in breast cancer and adjacent normal tissues by immunohistochemistry Negative expression of PARP1(**A**), BRCA1(**B**) and BRCA2 (**C**) in the same adjacent normal breast tissues; Negative expression of PARP1 (**D**), BRCA1 (**E**) and BRCA2 (**F**) in the same breast invasive ductal carcinoma (Stage IIa, TNM: T2N0M0); Positive expression of PARP1 (**H**), BRCA1 (**I**) and BRCA2 (**3G**) in the same breast invasive ductal carcinoma (Stage IIIb, TNM: T4N2M0). Dark brown color indicated strong positive immunostaining. (Magnification X400).

To determine the prevalence and clinical significance of PARP1, BRCA1 and BRCA2 in breast cancer development, we investigated their expression in 110 cases of tissues and adjacent normal tissues by using immunohistochemistry (Table 2). The adjacent normal breast tissues were negative for expression of PARP1, BRCA1 and BRCA2 (Figure 3A–3C); Negative expression of PARP1, BRCA1 and BRCA2 in the same breast invasive ductal carcinoma (Stage IIa, TNM: T2N0M0) were showed as Figure 3D–3F. As demonstrated in Table 2, 35.0% (35/100) breast cancer tissues were positive for PARP1 expression in the nuclei (Figure 3H); 34% (34/100) breast cancer tissues were positive for BRCA1 expression in both the nuclei and cytoplasm (Figure 3I), 33% (33/100) breast cancer tissues were positive for BRCA2 expression in both the nuclei and cytoplasm (Figure 3G). The expression of both PARP1 and BRCA1, both PARP1 and BRCA2, or both BRCA1 and BRCA2 in breast cancer were 17%, 22%, and 15%, respectively. The expression of PARP1, BRCA1 and BRCA2 was not correlated with cancer patients' age, TNM Stage, and pathological pattern of cancer. In addition, the expression of PARP1 was correlated with pathology grade of breast cancer (*P* < 0.05).

**Table 2:** Clinical characterization of the 110 cases breast cancer and adjacent normal tissue arrays and association expression of PARP1, BRCA1 and BRCA2 in breast cancer tissues

variable	No. (%)	PARP1 Positive	BRCA1 Positive	BRCA2 Positive	PARP1+BRCA1 Positive	PARP1+BRCA2 Positive	BRCA1+BRCA2 Positive	All Positive
All patients	110	35	35	33	17	22	15	10
Cancer	100	35 (35%)	34 (34%)	33 (33%)	17 (17%)	22 (22%)	15 (15%)	10 (10%)
Normal	10	0 (0%)	1 (10%)	0 (0%)	0 (0%)	0 (0%)	0 (0%)	0 (0%)
	*P* value	0.056	0.231	0.070	0.337	0.214	0.404	0.637
Age at diagnosis (cancer patients, *N* = 100)								
≤ 36	8	2	1	3	1	1	0	0
>36	92	33	33	30	15	22	15	10
	*P* value	0.817	0.342	1.000	1.000	0.766	0.470	0.712
TNM stage at diagnosis (*N* = 100)								
I	14	4	5	3	2	2	2	2
II	68	26	22	23	11	16	10	6
III + IV	18	5	7	7	4	4	3	2
	*P* value	0.613	0.864	0.562	0.797	0.731	0.976	0.824
Pathology Grade at diagnosis (*N* = 100)								
1	26	6	8	10	2	4	4	1
2	67	29	23	22	15	18	15	9
3	7	0	3	1	0	0	0	0
	*P* value	0.025	0.121	0.482	0.110	0.168	0.307	0.253
Pathological Pattern of Cancer (*N* = 100)								
−		65	66	67	*P* = 0.951			
+		25	20	22				
++		6	12	10				
+++		4	2	1				

## DISCUSSION

The concept of synthetic lethality is clinically translatable, which is the efficacy of drugs that target the single-strand DNA repair enzyme poly (ADP-ribose) polymerase (PARP) in tumors with mutations in the BRCA1 and BRCA2 genes. So far, only PARP1 and BRCA1/2 are single synthetic lethal interaction which has been shown to be therapeutic promise. Although clinical phase 1 and 2 studies of the synthetic lethal interaction have been established in breast, ovarian or prostate cancers, all trials about synthetic lethal therapies were not successful in the end [14]. The reason why clinical phase 1 and 2 studies of synthetic lethality were fail are extremely complicated. First, the major obstacle is that genetic and pharmacological perturbations do not always have the same functional outcome. Second, the context will impact synthetic lethality in human cancer cells. As we know, *in vitro* studies with cell culture do not directly address the role of the tumor microenvironment, and that cellular features and function may differ from cell line to cell line. Genetic modifiers can serve as biomarkers to predict which tumors are most likely to display the synthetic lethal interaction. Third, synthetic lethality interactions are now only being studied for their therapeutic potential and are designed to target the specific genetic and epigenetic phenomena associated with tumor formation, and thus are predicted to be highly selective [15]. It is currently unclear how common this type of strong genetic interaction is in human cells [16]. Lastly, the synthetic lethal interaction in two genes or multiple genes are very complex especially in human cancer. Malignant transformation, driven by gain-of-function mutations in oncogenes and loss-of-function mutations in tumor suppressor genes (including BRCA1 and BRCA2), results in cell deregulation that is frequently associated with enhanced cellular stress (for example, oxidative, replicative, metabolic and proteotoxic stress, and DNA damage) [17, 18]. Most cancer cells have at least two mutated genes before oncogenic transformation occurs [19, 20]. These drive genes include c-myc, p53, and EGFR et al. The interactions of synthetic lethal partners are different. For the single synthetic lethal interaction targeting tumor-suppressor genes when loss-of-function mutations drive tumorigenesis, BRCA2-PARP1 synthetic lethality is probably a hard synthetic lethality and the BRCA1-PARP1 synthetic lethality is slightly softer. Even synthetic lethal interactions can be found in non-oncogene and secondary gene mutation can be found in both cancer cells and cancer patients [21]. In fact, recent studies [22, 23] propose that EGFR and CDK12, other than BRCA1 and BRCA2 gene mutations, should be considered as a candidate PARP1/2 inhibitor response biomarker. The complexity of a human cell compared to a yeast cell may suggest that human cells display more redundancy and more resilient to perturbations. So the synthetic lethal interactions would be less frequent.

To our knowledge, the mechanism leading to autoantibody production in cancer was not completely understood. In this study, we focused only on the theme that immune response to tumor-associated antigens (TAAs) would result in the production of autoantibodies. Our previous studies have indicated that autoantibodies appearing with malignant transformation are more likely related to events associated with tumorigenesis and can be used as reporters identifying aberrant cellular mechanisms in tumorigenesis [24]. Further study on expression of novel TAAs to synthetic lethality in cancer could provide a potential mechanistic framework for the production of autoantibody against these TAAs. In this study, our results showed autoantibody responses to PARP1 abundantly expressed in breast, lung, ovarian, and liver cancers. We determined that elevated PARP1 expression in breast, lung, ovarian, and liver cancers has also been proposed as a predictive biomarker of PARP inhibitor response. A similar study was also reported by another group in lung cancer [6]. In addition, Immunohistochemistry study with breast cancer tissues also indicated that elevated PARP1 expression and pathology grade at diagnosis to PARP1 expression was different, which indicated the concordance of PARP1 status between serum and tumor tissue in breast cancer. Although autoantibodies responses to BRCA1 and BRCA2 abundantly expressed in breast cancer sera, Immunohistochemistry study with breast cancer tissues showed that the expression of BRCA1 and BRCA2 has no difference compared to normal breast tissues. The reason why expression of BRCA1 and BRCA2 are different in this study could be small sample size (especially normal tissue specimens) and heterogeneity between serum and tissue. The striking difference was autoantibody responses to PARP1 in prostate cancer were very low percentage. The reason for this low percentage may be due to the different profiles of autoantibody appeared in different cancers. In addition, the concordance of drive gene status between peripheral blood samples and matched tumor tissues has been reported to be varying from 59.1% to 92.0% [25], which is consistent with the observation that cancer heterogeneity in drive gene expression, mutation, or amplification has been extensively found in most types of cancers [26].

Recent emerging evidence indicate the elevated PARP1 expression in lung cancer. To prove the finding that the synthetic lethal interactions were also existed in lung cancer, we have tested the autoantibodies to PARP1and BRCA1/BRCA2 in sera from lung cancer patients. This high prevalence of autoantibody to PARP1 (22.4%) was notable, and meanwhile there was very low frequency of autoantibodies to BRCA1 (4.3%) and to BRCA2 (1.1%) in lung cancer sera. The reason for this difference might be that there was no synthetic lethal interactions between PARP1 and BRCA1/BRCA2 in lung cancer or there might be existed other types of synthetic lethal interactions, which is supported by recent studies [22, 23]. Our studies further suggest that synthetic lethality interaction (PARP1 and BRCA1/BRCA2) only exist in certain types of cancers.

In this study, our data showed a very high autoantibody response rate to BRCA1 (50.0%) but there was a quite low frequency of antibody to BRCA2 (5.9%) in ovarian cancer sera, which were consistent with a previous report that the estimated mutation rates for BRCA 1 and BRCA2 in ovarian cancer were ranged from 22%-65% and 10%-35%, respectively [27]; Some studies further reported that the BRCA2-PARP1 synthetic lethality is probably a hard type of synthetic lethality and the BRCA1-PARP1 synthetic lethality is slightly softer [6]. So it is plausible that the BRCA1-PARP1 synthetic lethality interactions are of frequent occurrence but the BRCA2-PARP1 synthetic lethality interactions are rare in ovarian cancer. Therefore, this might be the reason why clinical phase 1 and 2 studies of synthetic lethality in ovarian cancer were not successful. Collectively, our studies indicate that the softer combination of BRCA1-PARP1 synthetic lethality is not identified to lead to the occurrence of autoantibody responses in ovarian cancer.

Many studies have been interested in the use of TAAs or anti-TAAs autoantibodies as serological markers for cancer diagnosis, especially because of the general absence or a significantly lower frequency of these TAAs or anti-TAAs autoantibodies in normal individuals and in non-cancer conditions [28, 29]. This idea has been tempered by low sensitivity when individual antigen-antibody reactions were studied. Due to the fact that a particular type of cancer may involve multiple gene mutations, the discovery and validation of a universal and absolutely reliable biomarker for all types of cancer seems very difficult and impossible [30]. So we have assumed that the drawback can be overcome by using a panel of carefully selected TAAs and that different types of cancer may require different panels of TAAs to achieve the sensitivity and specificity required to make immunodiagnosis a feasible adjunct to tumor diagnosis. Our previous publication [12, 31] also indicated that TAAs mini-array seems to be supplementary serological markers for the diagnosis of cancer. Many of these antigen-antibody systems are not found to be useful in differentiating cancer and normal control. Some antigen-antibody systems may be unique to a particular type of cancer, and some may not. This study, has provided evidence that the occurrence of autoantibodies to TAAs (PARP1 and BRCA1/2) is not uncommon, and a characteristic feature is also the presence of multiple autoantibodies, suggesting that autoantibodies to synthetic lethal interaction (PARP1 and BRCA1/2) might act as a biomarker for diagnosis of cancer. Further investigation is needed to make a more solid conclusion.

Although the types of cancer and the patient sample size were limited, our results still have clinical significance because it highlights a promising way in using the concept of autoantibodies to proteins involving in the synthetic lethal interactions in cancer. One of the limitations of this study relating to synthetic lethality is the lack of blood and tissue samples from the same patients. Our previous studies [32, 33] have demonstrated that cancer sera contain antibodies that react with a unique group of TAAs and the types of cellular proteins that induce these autoantibody responses are quite varied. We recently reported that [7] intracellular proteins involved in carcinogenesis have been shown to provoke autoantibody responses although not all these proteins can induce autoantibody response in cancer and only some of these proteins can induce antibody responses. Most importantly, this study demonstrated there might be different profiles of autoantibodies in different cancers. This study further determined that elevated PARP1 expression in breast, ovarian, lung, and liver cancers has also been proposed as a predictive biomarker of PARP inhibitor response. Autoantibodies to proteins involving in the synthetic lethal interaction (PARP1 and BRCA1/2) might act as potential biomarkers for diagnosis of cancer.

In conclusion, this study is the first attempt to define the autoantibodies to synthetic lethal interactions (PARP1 and BRCA1/2) which would be useful in developing therapeutic targets for treatment of certain types of cancer. The immune response to TAAs is a repository of information on partners in the synthetic lethality paradigm. Synthetic lethality represents a new paradigm for cancer diagnosis and treatment.

## MATERIALS AND METHODS

### Serum samples

Sera from 131 patients with breast cancer, 94 with lung cancer, 34 with ovarian cancer, 107 with prostate cancer, 76 with liver cancer, and 41 with pancreatic cancer as well as 135 sera from normal individuals were obtained from the serum bank of The Cancer Autoimmunity Research Laboratory, The University of Texas at El Paso (Texas, United State). Of 483 cancer patients, 227 (47.0%) were male, and 256 (53.0%) were female. Age from 22 to 81 years old and Mean age was 56.5 ± 12.2. All cancer patients were histologically confirmed and were diagnosed according to the American Joint Committee on Cancer (AJCC). None of cancer patients had received treatment with chemotherapy or radiotherapy. Sera from 135 healthy controls were collected from adults during annual health examinations in people who had no obvious evidence of malignancy. These normal human sera were used as controls in the study. The study was approved by the Institutional Review Board of University of Texas at El Paso and collaborating institutions.

### Purified human proteins and antibodies

Three purified human proteins (PARP1, BRCA1 and BRCA2) were commercially available from Abnova (United State, Catalog number are H00000142-Q01, H00000672-P01 and H00000675-Q01, respectively). The gene descriptions of PARP1, BRCA1 and BRCA2 are poly (ADP-ribose) polymerase 1, breast cancer 1 and breast cancer 2, respectively. They are prepared by wheat germ expression system *in vitro*.

### Enzyme-linked immunosorbent assay (ELISA)

Serum IgG antibodies against PARP1, BRCA1 and BRCA2 were assessed by ELISA as described in our previous report [12]. In brief, purified recombinant proteins were individually diluted in phosphate buffered saline (PBS) to a final concentration of 0.5 μg/mL for coating Immunolon2 microtiter plates (Fisher Scientific, Houston, TX) overnight at 4°C. The human cancer serum samples diluted 1:100 were incubated with the antigen-coated wells for 2 hours, then incubated with horseradish peroxidase (HRP)-conjugated goat anti-human IgG (Caltag Laboratories, Burlingame, CA) as a secondary antibody diluted 1:4000 for 2 hours followed by washing with PBS containing 0.05% Tween 20 at room temperature. The substrate 2, 2′-azino-bis (3-ethyl-benzothiazoline-6-sulfonic acid) diammonium salt (ABTS) (Sigma, St.Louis, MO), was used as the detecting agent. The OD of each well was read at 405nm, and the cut-off value for determining a positive reaction was designated as the mean absorbance of the 135 normal human sera plus 3 standard deviations (mean+3SD). As described in previous studies, 8 normal human sera representing a range of 3SD above and below the mean of the 135 normal sera, was always used in each experiment and the average value of the 8 normal sera was used in each run to normalize all absorbance values to the standard mean of the entire 82 normal samples. In addition, all positive sera were confirmed with repeat testing, as were some negative sera.

### Western blot assay

Positive serum samples tested by the ELISA method were further confirmed by Western blotting. Three purified proteins (PARP1, BRCA1 and BRCA2) were eletrophoresed by 10% SDS-PAGE and subsequently transferred to nitrocellulose membranes (Amersham Biosciences). Nitrocellulose membranes were cut in strips and the individual strips were pre-blocked in PBS containing 0.05% Tween 20 (PBST) with 5% non-fat milk for 2 hours at room temperature, then incubated for 2 hours with patients sera diluted 1:100, and finally incubated with HRP-conjugated goat anti-human IgG diluted 1:5000 for 1 hour followed by washing with PBST solution. Positive signals were captured by autoradiography using chemiluminescence (Pierce Biotechnology, Rockford, IL) according to the manufacturer's instructions.

### Immunohistochemistry (IHC) with breast cancer and adjacent normal tissue arrays for expression of PARP1, BRCA1 and BRCA2 proteins

Three breast cancer and adjacent normal tissue arrays (UC Biomax, Catalog# BC081115) were purchased (including 10 cases from cancer adjacent normal breast tissues and 100 cases from breast invasive ductal carcinoma). Tissue array slides were deparafinized with xylene and dehydrated with ethanol. Antigen retrieval was performed by microwave heating methods in TrilogyTM pretreatment solution for 20 minutes. Avidin/Biotin blocking solution was used to prevent nonspecific binding of antibodies. The sections were incubated with anti-BRCA1 antibody (1:100 dilution, US Abcam, ab16780), anti-BRCA2 antibody (1:100 dilution, US Abcam, ab27976), and anti-PARP1 antibody (1:1000 dilution, US Abcam, ab6079) for 1 hour at room temperature respectively. HRP Detection System (Cell Marque, Cat#951D-10) and DAB Substrate Kit (Cell Marque, Cat#957D-10) were used as detecting reagents. After counterstaining with hematoxylin (Cell Marque, Cat#930B-02), the sections were dehydrated and mounted. The slides were observed by light microscopy.

All sections were scored independently by three experienced pathologists. Based on the intensity of immune staining and the quantity of stained cells, the intensity of staining was arbitrarily graded as: absent (0), weak (1+), moderate (2+), strong (3+). The percentage of stained cells was use d to quantify the react ion as negative (0% of positive cells), 1+ (<10% positive cells); 2+ (10–50% of positive cells); 3+ (51–80% of positive cells); 4+ (>80% of positive cells). The final value of the analysis of each tissue sample was then expressed as an absolute value through the obtained score by multiplying the two individual scores (i.e., intensity of staining score times the percentage of stained cells score), then generates a final score ranging from – (no expression) to + (weak expression), ++ (moderate expression), or +++ (strong expression). Examples of scoring according to staining intensity and the percentage of stained cells are shown in Figure 3.

### Statistical analysis

The mean OD value of each group of patients' sera was assessed using a standard Mann-Whitney U test. The frequency of autoantibody to TAAs in each group of patients' sera and the expression profile of either PARP1 and BRCA1 or PARP1 and BRCA2 in cancer and healthy controls were compared using the Chi-square (X^2^) test with Fisher's exact test. A probability value less than 0.05 and 0.01 was considered statistically significant.
